# Residency training amid the COVID-19 pandemic: exploring the impact on mental health and training, a lesson from Iran

**DOI:** 10.1186/s12909-021-03029-4

**Published:** 2021-12-06

**Authors:** Ashraf Moini, Khadije Maajani, Ramesh Omranipour, Mohamad-Reza Zafarghandi, Ashraf Aleyasin, Roya Oskoie, Sadaf Alipour

**Affiliations:** 1 Breast Disease Research Center, Cancer Institute, Tehran University of Medical Sciences, Tehran, Iran; 2 Department of Endocrinology and Female Infertility, Reproductive Biomedicine Research Center, Royan Institute for Reproductive Biomedicine, ACECR, Tehran, Iran; 3 Department of Gynecology and Obstetrics, Arash Women’s Hospital, Tehran University of Medical Sciences, Tehran, Iran; 4 Department of Epidemiology and Biostatistics, School of Public Health, Tehran University of Medical Sciences, Tehran, Iran; 5 Department of Surgical Oncology, Tehran University of Medical Sciences, Tehran, Iran; 6 Department of Vascular Surgery, Sina Hospital, Tehran University of Medical Sciences, Tehran, Iran; 7 Department of Gynecology and Obstetrics, Shariati Hospital, Tehran University of Medical Sciences, Tehran, Iran; 8 Office of the Department of Surgery, Imam Khomeini Hospital, Tehran University of Medical Sciences, Tehran, Iran; 9 Department of Surgery, Arash Women’s Hospital, Tehran University of Medical Sciences, Tehran, Iran

**Keywords:** Coronaphobia, General surgery, Gynecology, Mental health, Obstetrics, Residency, Stress, Training

## Abstract

**Background:**

COVID-19 has affected the training programs and the clinical schedules of surgical wards in many countries, including Iran. Also, the continuous involvement with COVID-19 patients has caused stress in health care workers; among them, residents are on the frontlines of care delivery. Therefore, we designed a study to assess the mental effects of these circumstances, and the effects on General Surgery and Obstetrics & Gynecology residency training in the busiest surgical departments of our university.

**Methods:**

Participants of this cross-sectional study were residents of General Surgery and Obstetrics & Gynecology of Tehran University of Medical Sciences, and the conventional sampling method was used. We used a questionnaire consisting of 47 questions (mostly using multiple choice questions and answers on the Likert scale) about personal, familial, and demographic characteristics; training activities, and mental effects of COVID-19.

**Results:**

The response rate was 63.5%. (127 filled questionnaires). Around 96% of the residents had emotional problems, 85.9% were highly stressed about contracting COVID-19, 81.3% were worried about transferring it to their families; and 78% believed that their residency training had been impaired.

**Conclusion:**

Overall, our study shows the negative impact of COVID-19 on mental health and the training of residents. We propose that appropriate emotional support and suitable planning for compensation of training deficits is provided for residents.

## Introduction

The load of patients affected by COVID-19 and the large human workforce capacity that has been needed to overcome the conditions has been dealt with in different ways in largely affected countries. On one hand, disease detection and triage of patients to decrease the workload of hospitals have taken place [1]. For example in Italy, physicians have been recruited from other areas, and nursing trainees have been graduated sooner; in China, health workers have been transferred to Wuhan from all other cities; in England, medical students have been called to help in managing the epidemic conditions [2]. In Iran, some medical centers have been dedicated to COVID-19 management [3], and all medical specialties and sub-specialties in these hospitals have been re-distributed to deal with this infectious disease. Along with these modifications, the programs and schedules of residents of many clinical specialties have also changed. The characteristics of residency training in Iran are dependent on their specialty but are very similar in hospital-based settings. Normally, residency training in surgical specialties is mainly pursued in clinical settings. Seeing patients, approaching their complaints, appropriate clinical decision making about diagnostic and treatment plans, performing the appropriate surgery with the best technique, patient care in the postoperative period, and suitable follow-up of surgical patients are skills that residents have to acquire during their training. Therefore, their education is based on a calm sound environment where both emergency cases and elective patients are seen and managed by the residents, under complete supervision of their professors. During the years of residency of surgery, candidates gradually participate in more complex surgeries and obtain increasing autonomy and accountability. Even in ordinary conditions, because of the workload and irregular training time schedules, residents are far from their families for long periods [4].

During the recent pandemic, two main problems have affected surgery trainees: First, nearly all the learning opportunities have been interrupted: due to the shortage of hospital resources, and to maintain social distance, the flow of patients to elective clinics has dropped significantly. Also, many elective operations have been cancelled. Second, at teaching hospitals, residents have been asked to change their shifts and schedules to compensate for the deficiency of health care professionals, and provide a workforce for attending COVID-19 patients [3, 5]. Therefore, two main issues have occurred after COVID-19: the residents have been both exposed directly to the dangers of COVID-19, and their learning opportunities have been modified.

COVID-19 conditions have affected both COVID-affected patients and healthy people all over the world in many aspects, including emotionally [6–9]; emotional consequences derived from these circumstances have been termed as “Coronaphobia” [10]. Health care workers continuous challenge in the frontline of the anti-COVID battle put them at higher risk [11], leading to serious consequences of the present situation on the mental health of healthcare workers [12, 13], based on probable risk factors [14, 15]. Moreover, world pandemics that have occurred in the past have been associated with different mental disorders such as anxiety, depression, exhaustion, and post-traumatic stress in health care workers [16].

These occur in all groups of medical workers, and in all levels of physicians who are in contact with affected patients.

In residents’ lives, the normal flow of work has been disturbed abruptly, and in addition a component of fear has been imposed on them; these could lead to both physical and mental collapse [17]. Also, considering the direct and first-line contact of residents with COVID-19 patients, the situation could impose emotional disruptions on them.

We hypothesized that these consequences of the COVID-19 pandemic would have a significant negative impact on the surgical residency training, and the stress caused by the outbreak would cause a substantial emotional burden on these residents. General Surgery (GS) and Obstetrics and Gynecology (OG) residents are a major component of our country’s healthcare workforce in university hospitals because of the very large number of trainees in these two specialties relative to most other specialties. Also, residents of GS and OG were faced with an extreme workload not only because of the usual frequency of emergency cases in these specialties but also in order to cover COVID-19 patients; whether for diseases related to GS or OG as a surgeon or for COVID-19 itself as a general practitioner. Tehran University of Medical Sciences (TUMS) is the largest Medical University in Iran, with the highest number of residents and fellows; and its hospitals are referral for other hospitals in the city and the whole country. Also, Tehran, the capital of Iran, was among the first and most affected cities of the country affected by COVID-19. The number of residents of GS and OG is among the highest in comparison with other residencies, and regarding their number and their capacities, they were involved very much in COVID-19 situations. Therefore, we carried out a study to investigate the effects of dealing with COVID-19 cases on the mental health of the residents of these two busy surgical departments (GS and OG) in TUMS, and the impact on their work and education as perceived by the residents themselves.

## Materials and methods

The study protocol was approved by the Deputy of research of TUMS (Code 99-2-100-48,539), and the ethical approval was issued by the Ethics Committee of TUMS (Code: IR.TUMS.VCR.REC.1399.499). All methods were carried out in accordance with relevant guidelines and regulations. In this cross-sectional study, the study population consisted of all the residents of GS and OG of the training hospitals of TUMS; and the conventional sampling method was used. The only inclusion criteria were to be active in the hospital in the COVID era, and willing to participate. The exclusion criteria included being on a leave (for example, for giving birth to a baby) and not being prepared to fill in the questionnaire.

According to the approved protocol, a researcher-made questionnaire was prepared by holding several online meetings among the Director of the GS Department, the Director of the OG Department, two professors of each department, and two education experts from the two departments. The final questionnaire included 15 questions about personal, familial, and demographic characteristics as well as underlying diseases [18, 19]; ten questions about the conditions of the residency activities and pertinent activities during COVID-19; ten questions about the mental effects of COVID-19 based on the themes investigated and validated by Khodabakhshi et al. [20] and Abdessater et al. [21]; and 12 questions from the Persian version of the short General Health Questionnaire (GHQ-12). The GHQ-12 has been validated in Iran by Yaghubi et al., and the appropriate reliability of the Persian translation has a Cronbach’s of 0.92 [22, 23].

We planned to send the questionnaires to all residents of these two wards, and recollect as many filled questionnaires as possible. Therefore, we distributed the printed questionnaires and the informed consent forms at the same time among questionnaires after their exams. The questionnaire was handed to all the residents of GS and OG of TUMS from June to August 2020. Residents were requested to answer the last 12 questions by considering the changes they felt in each item due to the COVID-19 conditions. According to the standard GHQ-12, these consisted of 6 positive questions, for example enjoying daily activities; and 6 negative questions, like feeling sad or depressed. We used the 4-point Likert scale to rate the answers. In positive questions, the *a*, *b*, *c* and *d* choices in the answers were rated 3, 2, 1, and 0, respectively. In negative answers the sequence was reversed, so that choice *a* got zero and choice d was scored as 3. Therefore, the highest score would be 36. According to Yaghubi et al. [23], a cut-off point of 15 was defined (maximum specificity score of 95.8%), where scores less than 15 were considered healthy, and scores above 15 indicated disturbed COVID-related mental health status (CMH).

We used mean ± standard deviation (SD) to describe quantitative variables, and frequency (percent) to describe qualitative variables. The Chi-square test was used for assessment of the association of quantitative variables with CMH, and logistic regression was used to find the effect of variables such as sex, underlying disease, history of infection by COVID-19, underlying disease in family member, supplies of PPE, presence of professors, managing COVID-19 patients, practicing in COVID-19 wards on CMH. The significance level was considered *p* < 0.05 for all analyzes. All statistical tests were performed by SPSS 26.

## Results

Among the 200 distributed questionnaires, 127 were recruited; showing an answer rate of around 63.5%. The mean age of the responders was 30.9 ± 2.4 and most of them were male (61.4%). Table 1 shows the demographic and personal features, past history and COVID-19 history of the residents.

**Table 1:** Personal, familial and demographic characteristics and COVID-19 history of all residents (*N* = 127)

Variable		
Age (*N* = 125, 2 missing)	25-29	27 (21.1%)
	30-34	89 (69.5%)
	35-39	6 (4.7%)
	≥40	3 (2.3%)
Sex	Male	78 (61.4%)
	Female	49 (38.6%)
BMI (mean ± SD) 24.8 ± 3.4	< 18.5	5 (3.9%)
	18.5-24.9	67 (52.8%)
	25-29.9	50 (39.4%)
	≥30	5 (3.9%)
Underlying disease^a^	No	112 (88.1%)
	Yes	15 (11.8%)
Underlying disease^a^ in family members^b^	No	45 (35.9%)
	Yes	82 (64.1%)
Marital status	Single	62 (48.8%)
	Married	65 (51.2%)
Residency year	First-year	27 (21.3%)
	Second-year	32 (25.2%)
	Third-year	33 (26.0%)
	Fourth-year	35 (27.6%)
Smoking	Yes	14 (11.0%)
	No	113 (89.0%)
History of COVID-19 affection	No	91 (71.7%)
	Yes, based on symptoms	28 (22.0%)
	Yes, based on a positive test	8 (6.3%)
Having children	No	107 (84.3%)
	Yes	20 (15.7%)
Living with parents	No	96 (75.6%)
	Yes	31 (24.4%)

^a^Including diabetes mellitus, hypertension, ischemic heart disease or heart failure, respiratory disorders, immunosuppressive states, psychological disorders; ^b^ Including only members of the family who live with the resident; SD = standard deviation

We had missing data in some of the variables, the percent in the following results are calculated relative to the total number of answers (excluding missing data) in each item. We have added this explanation in the text. Regarding educational issues, 62 (51.2%) residents reported a significant decrease in the number of training operations. The COVID-19 pandemic has caused new arrangements in the work hours of professors to avoid crowding in hospitals and provide the possibility for COVID-19 wards to be visited by specialties for their surgical complaints. Therefore, we inquired residents about any change in the rate of supervision of their professors over their surgical activities in the COVID-19 era. Among all residents, 84 (70.5%) believed that the presence of their professors in the operating room had decreased, and 101 (85.9%) stated that their residency training had been impaired.

Residents had to attend to COVID-19 patients in two different ways: first, for prenatal care or management of surgical and gynecological diseases of patients affected by COVID-19, second as a general practitioner in compensation for the shortages in residents and specialists of infectious disease in the management of COVID-19 patients. Results of our study showed that 103 (80.5%) of the residents had to manage COVID-19 patients which were directly related to their field of residency, and 48 (37.5%) had to take care of COVID-19 patients that were not related to their field. Seventy-four of the residents (61.1%) stated that they had been instructed about the suitable approach to COVID-19 patients, and 87 (71.9%) declared that they could appropriately manage these cases. Among them, only 74 (61.7%) stated that appropriate personal protective equipment (PPE) was provided for them by the hospital.

None of the residents had a previous history of mental disorders. However, the questionnaires showed that 123 (96.1%) experienced emotional problems in the COVID-19 circumstances. Fifty-six residents (45.5%) reported being highly stressed about the possibility of getting affected by the disease, while 100 (81.3%) were worried about transferring the virus to their families. In addition, 54 residents (43.9%) had become excessively obsessed with cleaning and disinfecting themselves and their clothes. The worries of the residents about themselves and their family were not related to their affection with COVID-19 (*p* = 0.56), their underlying disease (*p* = 0.62), smoking history (*p* = 0.59), their presence in the COVID ward (*p* = 0.65), the PPE supplied by the hospital (*p* = 0.87), and the physical health status of their family (*p* = 0.91); further details are demonstrated in Table 2.

**Table 2:** Relation between COVID-related mental health status and surveyed variables (chi-squared tests)

Variable	CMH positive, N (%) Total = 122 (96.1)	CMH, Negative, N (%) Total = 5 (3.9)	Chi2	*P*-value^a^
Affection with COVID-19				
Yes	34 (97.1)	1 (2.9)	0.16	0.68
No	86 (95.6)	4 (4.4)		
Underlying disease				
Yes	16 (100.0)	0.0	0.75	0.38
No	106 (95.5)	5 (4.5)		
Smoking History				
Yes	14 (100.0)	0.0	0.64	0.42
No	108 (95.6)	5 (4.4)		
Presence in COVID ward				
Yes	94 (95.9)	4 (4.1)	0.02	0.67
No	28 (96.6)	1 (3.4)		
PPE supplied by the hospital				
Yes	79 (96.3)	3 (3.7)	0.04	0.58
No	43 (95.6)	2 (4.4)		
Family’s physical health status				
Yes	77 (95.1)	4 (4.9)	0.59	0.65
No	45 (97.8)	1 (2.2)		

^a^ Fisher exact test *p*-value. CMH = COVID-related mental health status

As shown in Table 3, results of univariable and multivariable Logistic regression analysis did not show any statistically significant association between CMH and demographic or other education-related variables in the questionnaire.

**Table 3:** Univariable and multivariable logistic regression according to the independent variables in the questionnaire

	Univariable			Multivariable		
Variable	β (SE)	P-value	Unadjusted OR (95%CI)	β (SE)	P-value	Adjusted OR (95%CI)
Sex	−0.96 (1.11)	0.45	0.39 (0.04-3.56)	−2.28 (1.78)	0.19	0.10 (0.00^a^-3.30)
Underlying disease	17.13(8.20)	0.17	–	−18.96 (8.90)	0.10	–
history of affection by COVID-19	0.46 (1.12)	0.58	1.32 (0.16-15.20)	1.14 (1.50)	0.44	3.14 (0.16-59.80)
Underlying disease in family member	−0.81 (1.12)	0.37	0.42 (0.40-3.50)	−0.75 (1.40)	0.59	0.47 (0.03-7.32)
supplies of PPE	−0.23 (0.83)	0.62	0.75 (0.11-5.50)	−1.75 (1.27)	0.16	0.17 (0.10-2.10)
presence of professors	−1.21 (0.96)	0.39	0.46 (0.09-2.80)	−1.35 (1.24)	0.27	0.25 (0.02-2.95)
managing COVID-19 patients	0.68 (1.21)	0.55	1.79 (0.19-19.20)	- 0.09 (1.52)	0.95	0.9 (0.04-17.80)
practicing in COVID-19 wards	0.11 (1.17)	0.87	1.11 (0.11-12.20)	1.21 (1.60)	0.46	3.37 (0.12-88.80)

^a^0.003. CMH = COVID- related mental health

## Discussion

**Fig. 1:**
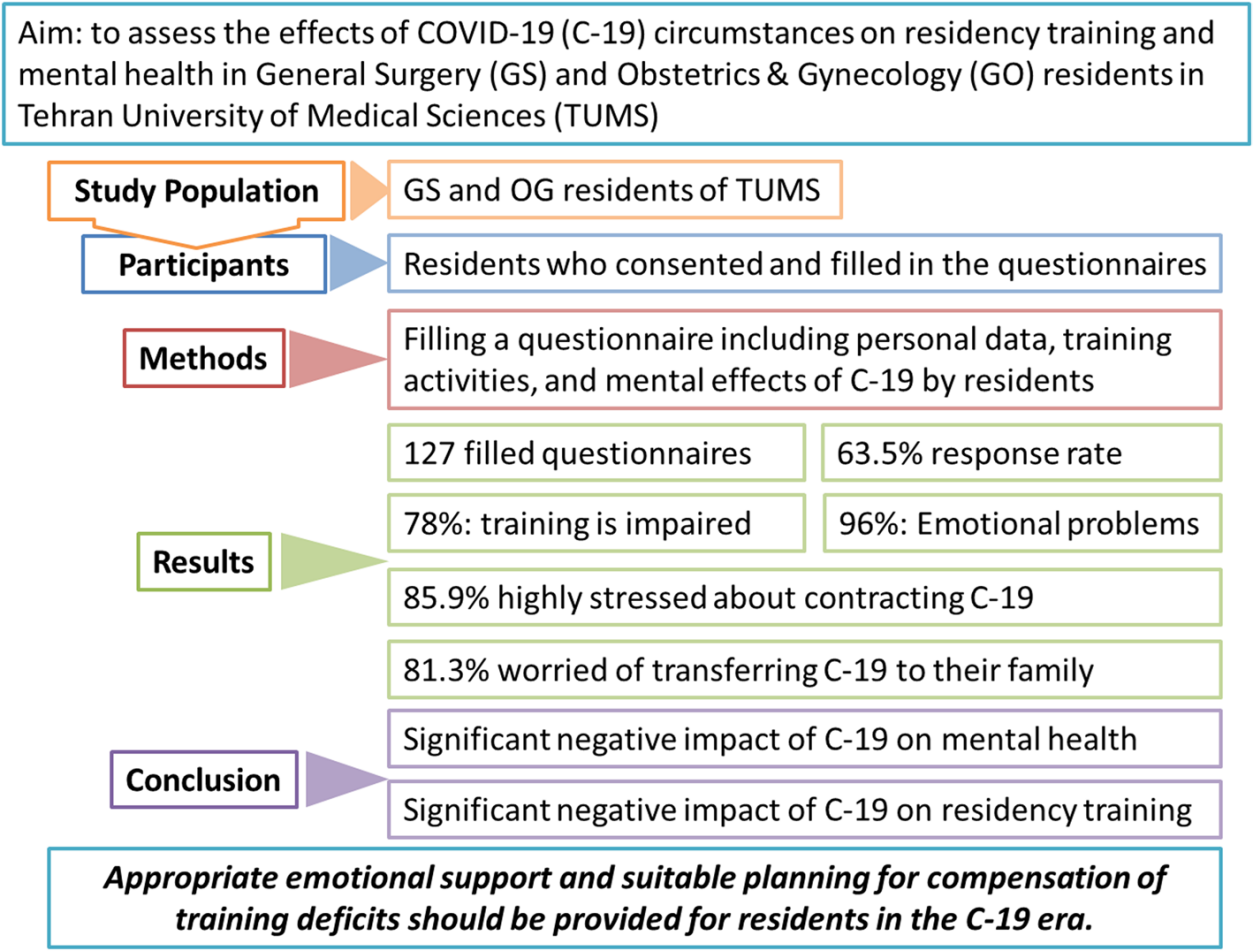
Summary of study and findings

We performed a study to investigate the conditions of residency training as well as the CMH of GS and OG residents during the COVID-19 pandemic and find out a high level of changes in both training and mental health status (Fig. 1).

The rate of answering was 63.5% in our study, which is an acceptable rate in comparison with other studies; response rates were from 30.4, 51, 55.5, and 73.7% in the studies of Brito et al. [24], Bitonti et al. [25], Abdessater et al. [21], and Collins et al. [4], respectively.

### Impact on residency training

Aziz et al. [5] studied 1102 GS residents in the USA and detected a significant negative impact on surgical training of the residents. Osama et al. [17] assessed the subject in 112 surgery residents of a tertiary care hospital in Pakistan, a high percentage of whom reported impaired surgical training during the pandemic. Collins et al. [4] surveyed residents of GS and plastic surgery in the USA and found that residents were mainly worried about their training during the outbreak. In our study also nearly 86% of the residents reported impairment in their training.

Brito et al. [24] investigated the status of OG residents in Brazil, and found that nearly a third were assigned to activities not related to their field of study; this occurred also for around 38% of our residents.

### Residents’ concerns

Residents in the study Aziz et al. [5] were worried about themselves or their families contracting the disease. He et al. [2] examined the concerns of GS residents about the pandemic in the USA, and detected that they were mostly worried about getting COVID-19, transferring the disease to their families, or the patients. Collins et al. [4] surveyed their participants (residents of GS and plastic surgery in the USA) regarding anxiety at the time of COVID-19, and showed that they had significant anxiety about their families and self-health. Also, a major concern of the surgery residents in the study of Osama et al. [17] was about their family’s health, and then fear of death from COVID-19. In our study also three fourth of the trainees were worried about transferring the disease to their family, while less than half were afraid about the infection of their selves.

### PPE use and preventive protocols

Around a quarter complained about defective supplies of PPE in the study of Brito et al. [24], while nearly half of the trainees in the study of Bitonti et al. [25] had been provided with adequate PPE. This rate was 38.3% in our study, which shows worse conditions in our hospitals in this regard. However, the definition of adequate PPE might be different in various cultures.

Bitonti et al. [25] stated that 80% of their residents had been instructed about preventive protocols, and two- thirds of the residents in the study of Brito et al. [24] had got instructed about the management of COVID-19 cases; these rates were not much different in our study (around 61%).

### Affection with COVID-19

Bitonti et al. [25] studied 476 OG residents in Italy and found out that around 6% had tested positive for COVID-19; these rates were very near the rate of positive COVID-19 tests in our residents (7.4%).

### Mental health status

We could not find any study that measured the mental health status of clinical residents during COVID-19 via standard questionnaires. However, previous studies have investigated the issue in health care workers. Amin et al. [11] assessed the psychological health of 250-medical professionals who were caring for quarantined COVID-19 patients in Pakistan and found a significant negative impact. However, in a study by Magnavita et al. [12] on health care workers in Italy who had been monitored for their mental health status before the recent pandemic, no increase in depression and anxiety rates were shown in the COVID-19 times. Meanwhile, a systematic review by Muller et al. [16] about medical human workforces showed sleep disturbance, depression, anxiety, and distress during the COVID-19 pandemic.

As we aimed to assess the conditions in residents, we measured the mental health status of the participants by using the GHQ-12 questionnaire; and detected a significant impact of the COVID-19 circumstances on the emotional status of our residents.

### Strengths and limitations

Existing studies asked the residents about their worries and anxiety related to their health and that of their families, or about defective training; but none of them examined the mental health status of the residents during COVID-19 by validated methods. In our study, similar to the previous ones, trainees were asked about their specific concerns about their and their family’s health status regarding COVID-19 infection, and also about the impairment of their training. But, most importantly, the mental health status of residents during COVID-19 was investigated by an international validated questionnaire for the first time (as far as we know); therefore the use of GHQ-12 in our research was a novelty among these types of studies. As an important contribution to the existing body of knowledge, its use yielded standard results confirming high levels of stress and emotional affection in the residents. To our knowledge, no similar study has been performed on residents in Iran, and studies in other countries have not considered the training conditions and mental health status of the residents as we did.

Our study had some limitations. Our sample size was small, and the response rate of participants was not high. Considering the novelty of the COVID conditions, we had no valid pre-defined questionnaire which could cover all the aspects of our study, and we thus had to design a questionnaire. Also, because many residents had not written the names of their hospitals and the questionnaires were anonymous, we could not analyze the results according to the hospital where they were active. Some participants might have answered hurriedly or by discussing with others, causing potential bias in the results. Lack of study baseline inevitably limited our observation of COVID-19 effects, we did not have the data about the mental health of the residents before COVID-19. On the other side, only residents of TUMS were entered in the study, generalization of results to every resident in the country might not be correct.

## Conclusion

The investigations in other countries showed a decrease in training opportunities, which is fairly expected in the COVID-19 era. Our study also shows the negative impact of COVID-19 on the training of GS and OG residents, both from their own point of view and when considering the changes in training opportunities. In addition, emotional problems arising in the residents even without any previous related underlying factors have been shown in this study. Emotional consequences or Coronaphobia were very prevalent in our small population of GS and OG residents. A main concern of the residents in this regard was the possibility of transmitting the viral disease to their families. The preparedness of the residents for managing COVID patients, although unrelated to their field of study, and their approval of the pertinent instructions also was detected. We recommend serious planning in order to provide adequate emotional support for residents during the present circumstances and suitable arrangements by the academic staff to compensate for the current deficit in residency training.

## Data Availability

Data and materials are in SPSS format and are in possession of the authors; they are available by request from the corresponding author at sadafalipour@yahoo.com.
